# 

**Published:** 1900-06

**Authors:** 


Secretary.

### PENNSYLVANIA STATE VETERINARY MEDICAL ASSOCIATION.

Secretary Ranck has just issued the following important letter in connection with the coming semi-annual meeting:

The semi-annual meeting of the Pennsylvania State Veterinary Medical Association will be held at Erie, on September 18, 1900. The object of this meeting is, as you know, for the advancement of the profession in our State, and as we are in advance of all other States in the country in the control and suppression of diseases, there is no reason for us to be backward in general association work.

There are a great many eligible men who would be benefited by becoming members, and would also be a decided help to the Association. Will you kindly see your near-by colleague and present him with a copy of the By-laws, which you can obtain from the Corresponding Secretary if you do not have them on hand, and urge him to join?

Since we expect to make this meeting a most successful one, and hope to have reports from all the committees, it will be of great benefit and importance for you to be present.

The programme promises to be very interesting and instructive, which I hope will be strengthened by hearing from you either in form of paper or reports of cases. Please notify me at your earliest convenience whether you will be present, also whether we can add your name to the programme.

E. M. RANCK,
Corresponding Secretary.

---

THE Veterinary Medical Association of New Jersey meeting in May was well attended, and much interest in Association work manifested. Additional strength was added in the election of several new members.

---

### PERSONALS.

The spring meeting of the State Board of Agriculture at Lock Haven, Pa., early in June, was addressed by State Veterinarian Pearson on "The Relation of the Wholesomeness of the Stable to the Health of Its Inmates."

Dr. H. D. Gill, of New York, moved into his new home and hospital quarters the latter part of May.

Dr. C. A. Mooney, of Hiawatha, Kansas, is employed by the English Government as veterinarian for the purchasing station at Kansas City.

The suppression of "Bovine Tuberculosis" is the title of a reprint from the annual report of the Rhode Island State Board of Agriculture, by Dr. Austin Peters, of Boston, Mass. Dr. Peters reviews the various plans suggested and comments upon their feasibility.

Continuing their splendid records as marksmen in military circles of New York State, Drs. William H. and Bryer Pendry, of the Borough of Brooklyn, never forget to train their guns Washingtonward in favor of army legislation.

Dr. J. R. Fountain Rochester, of Denton, Maryland, has taken a degree in human medicine in one of Philadelphia's medical colleges.

Dr. M. H. Reynolds, of St. Anthony Park, Minnesota, is one of the most extensive contributors to veterinary, agricultural stock raising, and sanitary publications in this country. He wields a busy pen.

"Nolan and his pet dog" is the way the boys put it at old "Penn." In the veterinary department, now some eighteen years old, he fills the rôle of "the only Nolan."

Dr. A. B. Kelly, formerly with his brother, Dr. W. H. Kelly, of Albany, N. Y., has located in Johnstown, same State.

Dr. James L. Robertson, of New York, and F. H. Mackie, of Fairhill, Md., acted as veterinarians to the second annual open-air horse show at Baltimore in May. Dr. A. W. Clement acted as veterinary referee.

Dr. W. L. Rhoads, of Lansdowne, Pa., is now the promoter of a wave motor, and in harnessing the wave force of the ocean he is sanguine of riding into billows of wealth.

Dr. George C. Beckett, of New York City, is no longer engaged in veterinary practice.

Dr. W. E. Martin, of Winnipeg, Manitoba, conducts a horseshoeing shop in connection with his practice.

Dr. L. A. Merillat, of Chicago, Ill., is one of the busiest practitioners of the Western cities. Active professional work, college teaching, association work, legislation halls, State board duties, and journalistic ambitions make strong demands upon his time and call for much labor, energy, and methodical work.

Dr. Francis Bridge, of Philadelphia, is extensively interested in the building up of one of the most popular residential sections of the "City of Brotherly Love."

Dr. J. C. Foelker, of Allentown, Pa., is a frequent visitor to the "Quaker City," and on one of his recent visits to this city was invited to dine at the University restaurant, so well known to every U. of Pa. boy the past ten years. He was so struck with the building's shape and interior arrangement that he gave it the lasting name of "Noah's Ark."

Dr. Alexander Glass, of Philadelphia, Pa., a member of the Corinthian Yacht Club, was among those who were struggling for club trophies with his yacht in the mile run on May 26th.

Dr. Thomas B. Rogers, of Woodbury, N. J., is preparing a dose-book and brochure of remedies indicated in the various diseases of the domesticated animals for one of Philadelphia's most progressive medical firms.

Editor Hoskins, of the JOURNAL, is basking in the realms of peace, while his successful adversary in the Mayoralty fight in the "Quaker City" is writhing under the scathing criticism of an enraged people. "Uneasy lies the head that wears a crown."

State Secretary Ridge, of Pennsylvania, has been one of the most thoroughgoing representatives of the A. V. M. A., and is a strong example of one who believes "what is worth doing is worth doing well."

Drs. Budd, Harker, and Runge, of New Jersey, visited Washington in May in the interest of the army veterinary bill. They represented the State Association and interviewed the Congressmen from that Commonwealth, seeking their support in the House.

Dr. Charles Lintz, of Chester, Pa., recently underwent a serious surgical operation for a long-standing condition. His recovery was prompt and the relief complete.

Dr. M. Jacob, Resident Surgeon of the Veterinary Department of the University of Pennsylvania, was a visitor to his former home in May. The frequency of these visits is said to excite suspicion and comment among the boys.

Dr. John J. Cattanach, of New York City, will fill the rôle of veterinarian to the Atlantic City, N. J., horseshow, July 11th to 14th, inclusive.

Dr. Oscar Seeley, of Philadelphia, will spend the summer at the Beechwood Inn, Jenkintown, Pa.

